# Quasi-static Analysis Based on an Equivalent Circuit Model for a CMOS Terahertz Plasmon Detector in the Subthreshold Region

**DOI:** 10.3390/s19071508

**Published:** 2019-03-28

**Authors:** Ju-Hee Son, Jong-Ryul Yang

**Affiliations:** Department of Electronic Engineering, Yeungnam University, Gyeongsan 38541, Korea; sonju@ynu.ac.kr

**Keywords:** equivalent circuit model, CMOS plasmon detector, terahertz detector, detector optimization, small-signal analysis, sub-threshold operation, quasi-static

## Abstract

An analytic method for a complementary metal-oxide-semiconductor (CMOS) terahertz plasmon detector operating in the subthreshold region is presented using the equivalent circuit model. With respect to design optimization of the detector, the signal transmission from the antenna port to the output of the detector is described by using the proposed circuit model, which does not include a complicated physical operating principle and mathematical expressions. Characteristics from the antenna port to the input gate node of the detector are analyzed through the superposition method by using the characteristic impedance of transmission lines. The superposition method shows that the effect of interconnection lines at the input is simplified with the optimum bias point. The characteristics of the plasmon detection are expressed by using small-signal analysis of the single transistor at the sub-threshold operation. The results of the small-signal analysis show that the unity gain preamplifier located between the detector core and the main amplifier can improve the detection performances such as the voltage responsivity and the noise equivalent power. The measurement results using the fabricated CMOS plasmon detector at 200 GHz suggest that the unity gain preamplifier improves the detector performances, which are the same results as we received from the proposed analytic method.

## 1. Introduction

Terahertz waves exhibit characteristics, such as high absorption for the water molecules, high reflectivity for conductive metals, and transparency for dielectric materials with non-polarity, which are useful in several industrial applications, including imaging systems [1,2]. A terahertz imaging system mainly consists of a terahertz source, a quasi-optical system for irradiating the output terahertz wave to the detector and the object, and a detector to illustrate the output voltage depending on the incident power of the terahertz wave [2,3]. The quality of the terahertz imaging system is determined by the responsivity and the noise equivalent power (NEP), which are characteristics of the terahertz detector. In industrial applications of the imaging system, it is important to realize a detector with high responsivity and low NEP [2,3,4]. Various terahertz detectors including a microbolometer and Schottky barrier diodes are examined to improve detection characteristics [5,6,7,8]. Recently, a highly sensitive detector based on the complementary metal-oxide-semiconductor (CMOS) process was proposed by considering integration with a signal conditioning block and control circuits fabricated in the same CMOS process and the importance of implementing a large scale focal plane array [9,10,11]. A CMOS plasmon detector that detects terahertz signals higher than the operating frequency of the CMOS process is a promising structure for an imaging system because of the implementation of the large-scale pixel arrays and integration with the signal conditioning blocks [12,13].

The operation of the plasmon detector is described with the potential difference in the channel of the transistor that varies proportionally to the magnitude of an incident terahertz wave. The physical behavior of the plasmon detector is explained in detail by using the resonance characteristics of the plasma wave generated in the channel by the terahertz input signals from the Dyakonov-Shur plasma wave theory [14]. The detector operation is represented by the non-quasi-static analysis that models the channel of the transistor as a two-dimensional electron gas [14,15,16,17]. With respect to the electronics viewpoint, the plasmon detector is considered as a resistive mixer that is operated by the variation in the channel resistance in the transistor that changes due to the input signals [18]. A non-quasi-static model considering the effects of parasitic capacitors and distributed self-mixing operation, and a quasi-static model that describes the low-frequency operation as a square-law power detector, are used in the operating analysis of the terahertz detector [19,20,21]. Extant studies physically and accurately describe the operating characteristics of the terahertz detector. However, they are not useful in terms of providing an intuitive understanding of designing a plasmon detector. The analysis of detector characteristics in the weak inversion region is necessary to determine optimum design conditions because previous studies indicated that the photoresponse of the detector is maximized at the bias condition of the subthreshold region of the detector [16,17,19,20]. Additionally, power combining efficiency and impedance matching between the antenna and detector should be considered important factors in the improvement of the detector performances [21,22]. 

This study shows a quasi-static analysis based on an equivalent circuit model of the CMOS plasmon detector to improve the detector performances. The equivalent circuit model consists of the superposition method that describes the relationship of the characteristic impedances including the effect of the transmission line length and small signal analysis method that simplifies the operation of the plasmon detection in the subthreshold region. The analysis using the proposed equivalent model makes it easy to understand the design specifications and detector structure to improve the performances of the plasmon detector. The theoretical analysis of the plasmon detector using the proposed circuit model is described in Section 2. Section 3 shows the design of two CMOS plasmon detectors to verify the performance improvement method presented in the proposed analysis results. The usefulness of the proposed analysis is discussed with performance comparisons between two detectors in Section 4. Conclusions are detailed in Section 5. 

## 2. Quasi-Static Analysis Using the Proposed Equivalent Circuit Model 

**Figure 1:**
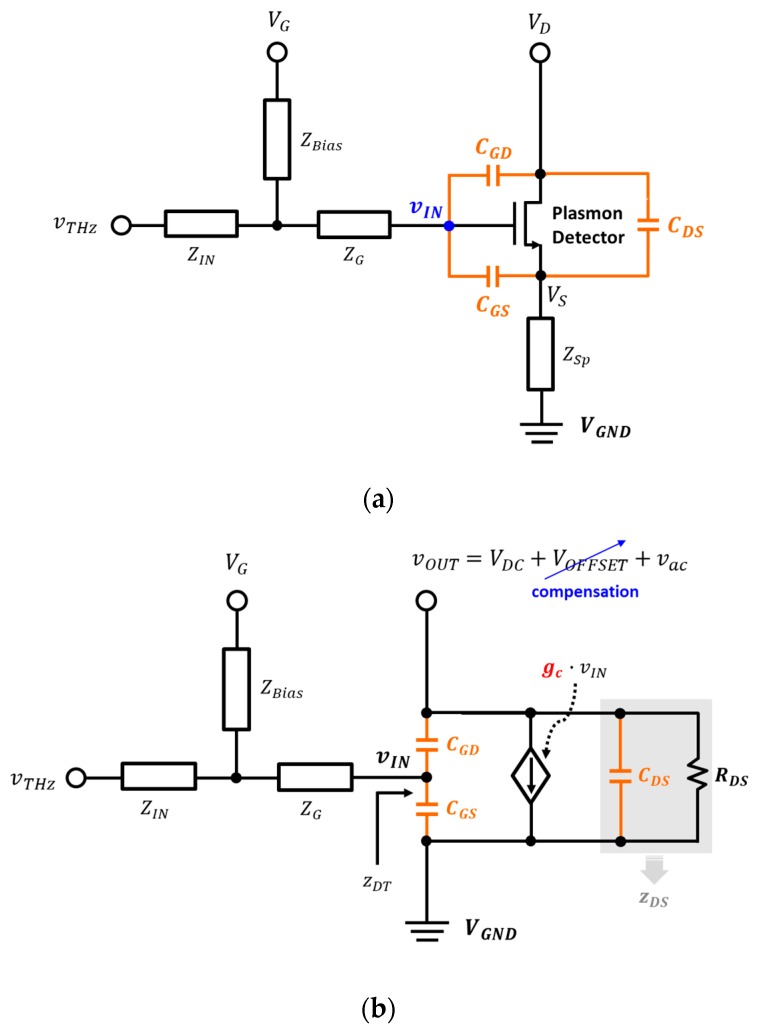
Schematic diagrams of the proposed model for the plasmon detector using the field-effect transistor (FET) at high frequency: (**a**) Basic model including the parasitic capacitances; (**b**) modified model for small-signal analysis.

The plasmon detector using a CMOS field-effect transistor (FET) was simplified with the basic configuration shown in Figure 1a. The gate node of the plasmon detector was directly coupled with the integrated antenna. The terahertz signal was received through the integrated antenna and transmitted to the gate of the detector device. The detector gate was also provided with an external gate bias voltage to control the modulation of the internal channel of the transistor [23]. The input signal $v_{IN}$ of the plasmon detector can be expressed with a terahertz input signal $v_{THz}$ and an external gate bias $V_{G}$ at high frequency as follows:

(1) $$v_{IN}=v_{THz}+V_{G}.$$

The detector input is connected to the input port of the integrated antenna and the external gate bias line through the transmission lines [24]. The transmission lines exhibit different electrical properties depending on the length of the transmission lines by the lumped-element model. The impedance of the transmission lines is modeled as the characteristic impedance considering the return loss and is modeled as constant impedance when the transmission line length is fixed. When the input port of the integrated antenna is connected to the gate of the detector device, the impedance from the integrated antenna to the point where the external gate bias line meets is expressed as $Z_{IN}$. The impedance from the external gate bias line to the point where the input port of the integrated antenna meets corresponds to $Z_{Bias}$. The impedance connected to the detector input from the point where the input port of the integrated antenna meets the external gate bias line corresponds to $Z_{G}$. The source of the detector device connected to the ground is ideally virtual ground, but a few offset voltages can be generated due to the wire bonding line. The characteristics of the interconnection line from the source of the device to the ground are denoted as the impedance $Z_{sp}$.

The detector device is modeled as a voltage source using a small-signal method as shown in Figure 1b because the terahertz signal that is applied to the detector input is extremely small. The current flowing at the detector device is determined by the detector input signal and transconductance. The relationship between the changes in the input voltage and output current of the detector at different frequencies is assumed as a plasmonic conversion transconductance $g_{c}$. The equivalent impedance of the detector device from the detector input corresponds to $z_{DT}$. The impedance $z_{DS}$ at the detector output node is represented by a parallel connection of the parasitic capacitor and load resistance and is expressed as

(2) $$z_{DS}=C_{DS}//R_{DS}=\frac{R_{DS}}{1+j\omega C_{DS}R_{DS}}.$$

The output voltage of the detector has an AC signal that the terahertz input signal is coupled through the parasitic capacitor $C_{GD}$ between the gate and the drain. The output node also detects DC signals based on the magnitude of the terahertz signal and generates a DC offset voltage by the structure of detector device [18]. The DC offset voltage has an effect on the output characteristics since the response to the terahertz input is shown in the DC voltage at the drain node of the detector. However, the proposed analysis neglects the DC offset voltage at the drain node, and it is assumed that the source of the detector device becomes ground.

### 2.1. Analysis Using a Superposition Method

**Figure 2:**
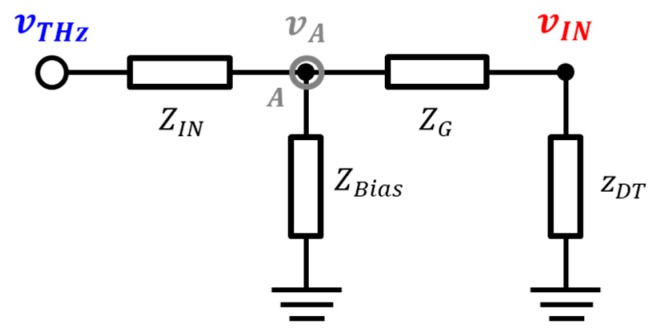
Schematic diagram by using the proposed superposition method at the input interconnection lines of the plasmon detector.

Impedance matching between the integrated antenna and detector input affects the performance of the detector. The superposition method does not provide a detailed impedance value although it can simplify the effect of the interconnect at the input of the detector device. The detector input has two independent voltage sources, namely the terahertz signal $v_{THz}$ which is an AC signal and the external gate bias $V_{G}$ which is a DC signal. The detector input is explained by the principle of superposition since the two input signals exhibit a linear relationship. Applying the superposition method for the ac voltage source, the external gate bias voltage source is considered as a short circuit as shown in Figure 2. The voltage of the input node $v_{IN}$ is expressed as (3) by the law of voltage distribution as

(3) $$v_{IN}=\frac{z_{DT}}{Z_{G}+z_{DT}}v_{A},$$

where $v_{A}$ denotes the voltage of the node A where the antenna input port meets the external gate bias line. The terahertz signal is represented by (4) using the Kirchhoff’s rules of current to node A. Substituting into (4) after modifying (3) for the voltage of node A, the terahertz signal is modified to (5) as follows:

(4) $$v_{THz}=\left(1+\frac{Z_{IN}}{Z_{Bias}}+\frac{Z_{IN}}{Z_{G}}\right)v_{A}-\frac{Z_{IN}}{Z_{G}}v_{IN},$$

(5) $$v_{THz}=\left[\left(\frac{Z_{G}+z_{DT}}{z_{DT}}\right)\left(1+\frac{Z_{IN}}{Z_{Bias}}+\frac{Z_{IN}}{Z_{G}}\right)-\frac{Z_{IN}}{Z_{G}}\right]v_{IN},$$

(6) $$v_{THz}=\left[\left(\frac{Z_{G}+z_{DT}}{z_{DT}}\right)\left(1+\frac{Z_{IN}}{Z_{G}}\right)-\frac{Z_{IN}}{Z_{G}}\right]v_{IN},$$

where $Z_{Bias}$ in (5) should be infinite to pass all the terahertz signal to the detector input, and the $Z_{IN}/Z_{Bias}$ term converges to zero and results in (6). The relationship between the input signal $v_{IN}$ of the detector and the terahertz signal $v_{THz}$ received from the integrated antenna is simply expressed as

(7) $$v_{IN}=M\cdot v_{THz},$$

where the parameter M denotes the reciprocal of the $v_{IN}$ coefficient at (6). The parameter M has a maximum to transfer all terahertz input signals to the gate node of the detector device. It should be satisfied as expressed in (8) to approximate the maximum value of 1 of the parameter M as follows:

(8) $$Z_{G}\ll z_{DT}.$$

Therefore, the transmission line corresponding to the $Z_{G}$ should be designed with a minimum possible length such that all terahertz input signal is applied to the gate of the detector device. 

### 2.2. Analysis Using a Small-Signal Method at The Subthreshold Region

A FET-based resistive self-mixing with a zero-biased channel is analytically derived for the drain current, which is represented in previous studies by the channel conductance and applied mixing signal for a single device of strong inversion ($V_{gs}>V_{th})$ for purposes of simplicity [19,25]. The time-independent drain current in the proposed detector model is derived based on previous studies as follows:

(9) $$i_{d}\left(t\right)=g_{c}\left(t\right)\cdot v_{IN}\left(t\right).$$

The plasmonic conversion transconductance $g_{c}\left(t\right)$ of the detector operating at strong inversion is approximated as follows:

(10) $$g_{c}\left(t\right)=k_{n}\left(v_{gs}\left(t\right)+V_{GS}-V_{th}-\frac{v_{ds}\left(t\right)}{2}\right),$$

where $k_{n}$ is the transconductance parameter of the FET. The parameter $k_{n}$ denotes the product of the process transconductance parameter $\mu C_{ox}$ and the aspect ratio of the transistor W/L where μ is electron mobility, $C_{ox}$ is the oxide capacitance, W and L are the channel width and length, and $V_{th}$ is the threshold voltage of the detector device. The self-mixing detector is simultaneously applied with the same AC signal to each port of the resistive mixer and all AC signals are equal [19]. The relationship between all ac signals and the terahertz input signal received from the integrated antenna is as follows:

(11) $$v_{THz}\left(t\right)=v_{gs}\left(t\right)=v_{ds}\left(t\right),$$

(12) $$v_{THz}\left(t\right)=V_{THz}\sin\left(\omega t\right).$$

Modifying (10) using (11) and (12) and substituting into (9), the current flowing through the drain node of the detector device is modified as

(13) $$i_{d}\left(t\right)=\frac{1}{4}k_{n}V_{THz}^{2}+k_{n}\left(V_{GS}-V_{th}\right)V_{THz}\sin\left(\omega t\right)-\frac{1}{4}k_{n}V_{THz}^{2}\cos\left(2\omega t\right),$$

where the first term denotes the DC component current, and the second and third terms denote the ac component current. All ac currents at the output node are filtered by the RC filter of the drain node that consists of the resistor $R_{DS}$ and parasitic capacitor $C_{DS}$. Only the dc current is extracted as

(14) $$I_{D}=\frac{1}{4}k_{n}V_{THz}^{2}.$$

The performance of the detector is predicted from the detected DC output current. The voltage responsivity $R_{V}$ and noise equivalent power NEP are indicators of the detector performance and are expressed as follows:

(15) $$R_{V}=\frac{I_{D}}{P_{in}}\cdot \frac{1}{g_{c}}=\frac{R_{in}}{4\left(V_{GS}-V_{th}\right)},$$

(16) $$NEP=\frac{\sqrt{N_{v}}}{R_{V}}=\frac{\sqrt{4KT/g_{c}}}{R_{V}}=\sqrt{\frac{4^{3}KT\left(V_{GS}-V_{th}\right)}{k_{n}\cdot {R_{in}}^{2}}},$$

where the input power $P_{in}$ is $V_{THz}^{2}/R_{in}$, and $N_{v}$ denotes the noise spectral density. The transconductance $g_{c}$ given only dc term by the ac signals filtered from the output node is $k_{n}\left(V_{GS}-V_{th}\right)$. The voltage responsivity and NEP appear as a function of the gate-to-source voltage. The maximum voltage responsivity and minimum NEP are obtained as the gate-to-source voltage approaches the threshold voltage in a way similar to the theoretical analysis in previous studies.

**Figure 3:**
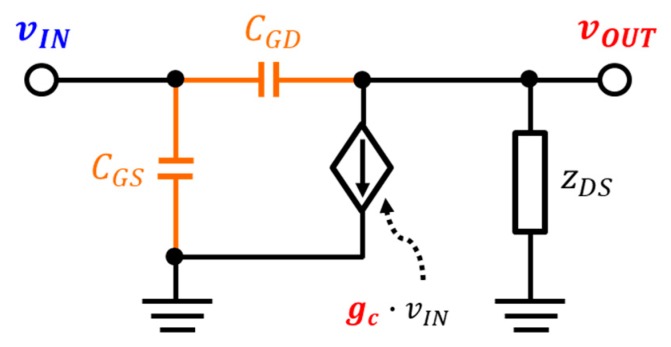
Schematic diagram of the proposed small-signal analysis for the plasmon detector core.

In contrast to conventional analysis methods, the detector device for the subthreshold region is analyzed by small-signal modeling as shown in Figure 3. The total current flowing to the detector device is obtained by applying Kirchhoff’s rules of current to the output node as follows:

(17) $$i_{total}=\frac{v_{IN}-v_{OUT}}{z_{GD}}=g_{c}\cdot v_{IN}+\frac{v_{OUT}}{z_{DS}}.$$

The voltage at the output node denotes the sum of the detected dc output voltage and AC voltage, where the AC voltage is the signal presented by the capacitor from the gate of the detector. The total output current is comprised of the current of the AC component and the DC component and is expressed as follows:

(18) $$v_{OUT}=V_{OUT}+v_{ac},$$

(19) $$v_{AC}=\alpha \cdot v_{THz},$$

(20) $$i_{total}=I_{DC}+i_{ac},$$

where the DC current in (20) is ignored because only a capacitor is present in the $z_{GD}$ and hence AC current flows at the total current. Substituting with the input voltage of (1) and the output voltage of (18) in (17), the total output current is modified as

(21) $$i_{total}=\frac{V_{GS}-V_{OUT}}{z_{GD}}+\frac{v_{THz}+v_{ac}}{z_{GD}}=g_{c}\cdot \left(v_{THz}+V_{GS}\right)+\frac{V_{OUT}+v_{ac}}{z_{DS}},$$

(22) $$\left(1-\alpha \right)v_{THz}=g_{c}\cdot z_{GD}\cdot \left(v_{THz}+V_{GS}\right)+\frac{z_{GD}}{z_{DS}}\left(V_{OUT}+\alpha v_{THz}\right),$$

where the first term in (21) cannot exist because it is a dc voltage and is summarized as (22) using (19). The analysis for the detector operating in the subthreshold region assumes that the current of the detector device is a drain current of the weak inversion as shown in (23) [26]. The drain current in weak inversion can be transformed as (24) by Taylor’s series as follows:

(23) $$i_{D}=I_{s}\cdot exp\left(\frac{v_{IN}}{nV_{T}}\right),$$

(24) $$i_{D}=I_{s}\cdot \left(1+\frac{1}{nV_{T}}\cdot v_{IN}+{\left(\frac{1}{nV_{T}}\right)}^{2}\cdot v_{IN}\right),$$

where $I_{s}$ is a constant proportional to W/L, n is a nonideality factor, $V_{T}$ is kT/q. The plasmonic conversion transconductance for the weak inversion is expressed as a function of input voltage and obtained using (24) as follows:

(25) $$g_{c}=\frac{\partial i_{D}}{\partial v_{IN}}=I_{s}\cdot \left(\frac{1}{nV_{T}}+{\left(\frac{1}{nV_{T}}\right)}^{2}\cdot (v_{THz}+V_{GS})\right).$$

Substituting (22) with (12) and (25) and assuming that the AC voltage disappears by the filter, the output node is extracted only for the DC voltage as

(26) $$\left(\frac{1}{2}I_{s}\cdot {\left(\frac{1}{nV_{T}}\right)}^{2}\cdot V_{THz}^{2}+I_{s}\cdot \frac{1}{nV_{T}}\cdot V_{GS}+I_{s}\cdot {\left(\frac{1}{nV_{T}}\right)}^{2}\cdot V_{GS}^{2}+\frac{1}{z_{DS}}\cdot V_{OUT}\right)\cdot z_{GD}=0.$$

The signal detected on the output node by the detector device in (26) is obtained as

(27) $$V_{OUT}=-\left(\frac{1}{2}I_{s}\cdot {\left(\frac{1}{nV_{T}}\right)}^{2}\cdot V_{THz}^{2}+I_{s}\cdot \frac{1}{nV_{T}}\cdot V_{GS}+I_{s}\cdot {\left(\frac{1}{nV_{T}}\right)}^{2}\cdot V_{GS}^{2}\right)\cdot z_{DS}.$$

The voltage responsivity and NEP of the detector operating in the subthreshold region are expressed from (27) as follows:

(28) $$R_{V}=\left|\frac{V_{OUT}}{P_{in}}\right|=I_{s}\cdot R_{in}\cdot R_{out}\cdot \left|\frac{1}{2}\cdot {\left(\frac{1}{nV_{T}}\right)}^{2}+\frac{{\left(\frac{1}{nV_{T}}\cdot V_{GS}+\frac{1}{2}\right)}^{2}-\frac{1}{4}}{V_{THz}^{2}}\right|,$$

(29) $$NEP=\frac{\sqrt{N_{v}}}{R_{V}}=A\cdot \frac{V_{THz}}{R_{in}\cdot R_{out}\cdot V_{GS}},$$

where $z_{DS}$ corresponds to the output resistance $R_{out}$ and a constant $\sqrt{4KT/{I_{s}}^{3}\cdot {\left(1/nV_{T}\right)}^{3}}$ is replaced by A for simplification purposes. As shown in (28) and (29), the voltage responsivity and NEP are represented as a function of $V_{GS}$, similar to the analysis results in strong inversion. The NEP is associated with voltage responsivity and decreases when the voltage responsivity increases. The voltage responsivity exhibits the quadratic function form for $V_{GS}$ where the maximum voltage responsivity exists. Equation (28) does not mean that the voltage responsivity and the incident voltage of the THz signals are in inverse proportion because the output resistance in the plasmon detector depends on the incident power of the THz signals. The input and output resistance should be increased to improve the voltage responsivity. It is important to increase the output resistance to increase the voltage responsivity because the input impedance has limits to be improved at high frequency due to the parasitic capacitances at the gate input node. 

## 3. Two Plasmon Detectors 

The detector performance is improved by increasing the input and output resistance of the detector device as shown in the theoretical analysis results of Section 2. However, the input resistance cannot be increased due to impedance matching to transfer all terahertz signals to the detector input. The output resistance cannot also be increased because the output signals are difficult to acquire. Therefore, the output node of the detector device requires a buffer amplifier to collect the signals detected, as opposed to increasing the output resistance. 

**Figure 4:**
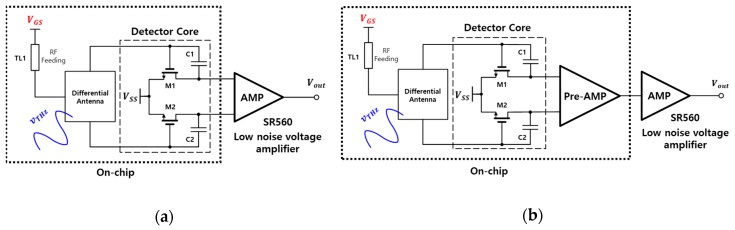
Architecture of the two THz detectors: (**a**) Differential detector directly connected to the external low-noise amplifier; (**b**) Detector including the subthreshold preamplifier.

Figure 4 shows two THz detectors as a proposed method to compare the performance of the detector when a buffer amplifier is added to the detector core to improve detector performance. The power delivered to the detector input is determined by the antenna area irrespective of the number of feeding lines in the antenna. The detectors in the proposed method use a differential antenna to remove harmonics generated at the detector and ignore the transmission line connected to the gate-to-source voltage, which is considered to be a parasitic antenna. The gate nodes of the detector cores M1 and M2 are connected with the integrated antenna by using the transmission lines on Metal 4 of the fabrication process. The interconnection from the antenna to the inputs of the detector cores is designed using 3-D electromagnetic-wave simulation. Figure 4a shows a differential detector structure operating at 200 GHz including the detector core and differential antenna with the output of the detector core connected to the external low noise voltage amplifier. On the other hand, Figure 4b consists of the same differential detector as in Figure 4a, but includes a preamplifier that operates in the sub-threshold region with a unity gain [27]. The preamplifier output is increased by the high transconductance of the preamplifier. The noise characteristic is also reduced by combining the outputs of the detector core. The outputs of the detectors in Figure 4 are compared by using the SR560 low noise voltage amplifier equipment. 

**Figure 5:**
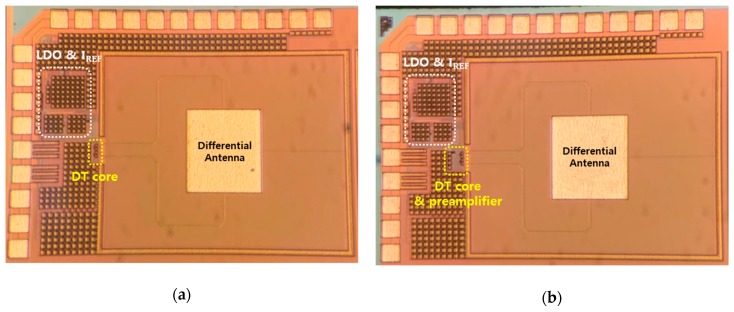
Photographs of the fabricated complementary metal-oxide-semiconductor (CMOS) plasmon detector integrated circuits: (**a**) Differential detector including the integrated antenna and the bias circuits; (**b**) Detector including the integrated antenna, the bias circuits, and the subthreshold preamplifiers.

The on-chip portions of the two THz detectors in Figure 4 are fabricated with a TSMC 0.25 µm mixed-signal CMOS process technology with one poly and five metal layers as shown in Figure 5. The chip area of the preamplifier with a voltage gain of 1 is 89.2 µm × 53.2 µm. The total area of the two detector circuits is 1.41 mm × 1.04 mm including the differential antenna and bias circuits that denotes the low drop-out (LDO) regulator and current reference circuit. The difference between Figure 5a,b is the presence or absence of a preamplifier, and all the remaining circuits are identical. The differential antenna integrated into the detector is designed and simulated using the three-dimensional electromagnetic wave simulation program. The simulation results obtained the S parameter of −31.5 dB at 200 GHz, an antenna bandwidth of 30 GHz and directivity of 4.9 dBi. 

## 4. Measurement Results and Discussion

### 4.1. Measurement Setup

**Figure 6:**
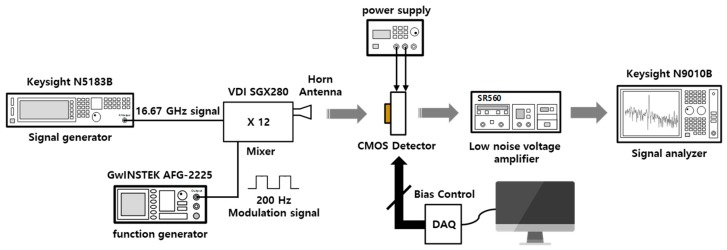
Measurement setup to obtain the detector performances.

The measurement setup is illustrated to obtain the responsivity of the detector in Figure 6. A 16.67 GHz signal with a power of 5 dBm from the signal generator is transmitted to the input of the mixer. The signal applied to the mixer input is multiplied by the multiplier corresponding to 12 of the mixer to output a 200 GHz signal. The amplitude of the THz signal is electrically modulated as opposed to the mechanical chipper by applying the pulse waveform with an amplitude of 5 V from the function generator to the Transistor-Transistor Logic (TTL) mode of the mixer. The frequency of the modulation signal is set as 200 Hz to easily obtain the detected signal in a measurement environment with DC offset and noise floor. The 200-GHz signal output from the mixer is radiated into free space through a horn antenna with 23 dBi gain. The input power received at the detector is calculated as −22.10 dBm given the distance from the antenna port to CMOS detector corresponding to 6.7 cm by the Friis transmission equation [28]. The output signal of the detector is connected to the 50-Ω input impedance of the low noise voltage amplifier. The signal amplified by the voltage gain of 49 dB in the low noise voltage amplifier is measured using a signal analyzer. The voltage gain of the low noise amplifier is set to the maximum gain that is obtained from the magnitude of the detector output signal. 

### 4.2. Performance Comparison Between two THz Detectors

**Figure 7:**
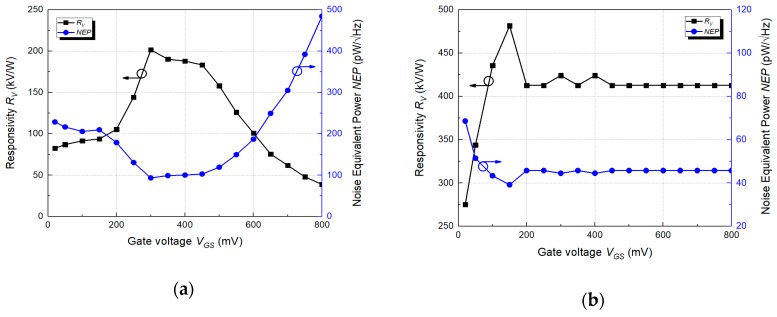
Measured voltage responsivity and noise equivalent power (NEP): (**a**) A detector without the preamplifier; (**b**) A detector including the preamplifier.

Figure 7 shows the voltage responsivity and NEP for gate bias voltage changes of the two detectors. The effective antenna area to obtain the responsivity was calculated to 5.51 × 10^−7^ m^2^ from the receiving area, the THz frequency, and the antenna gain [28]. Figure 7a illustrates the performance of the detector without a preamplifier. In the figure, the voltage responsivity increases as the gate bias voltage is increased and gradually decreases after the gate bias exhibits a maximum voltage responsivity of 202 kV/W at 0.25 V. This results in a graph similar to the quadratic function form of (28) in Section 2 and exhibits a gate bias voltage with the maximum voltage responsivity. The results also indicate that the detector operates in the sub-threshold region because the maximum voltage responsivity is observed when the gate bias voltage is lower than the threshold voltage level. Conversely, as shown in Figure 7b, the voltage responsivity of the detector with the preamplifier increased when the gate bias voltage increased, although it converged to a constant value by the operating region of the sub-threshold preamplifier. The detector exhibits the maximum voltage responsivity of 482 kV/W and is approximately 2.4 times the maximum voltage responsivity of the detector without the preamplifier. Figure 7 also shows the NEP for the detector by using the total noise spectral density measured using a signal analyzer. The NEP of the two THz detectors at the maximum voltage responsivity is minimized due to decreases in the noise around the signal, although the gate bias point is slightly different. The minimum NEPs of the two detectors in the measured results correspond to 130 pW/$\sqrt{\mathrm{Hz}}$ and 39.3 pW/$\sqrt{\mathrm{Hz}}$. The results reveal that the detector including the preamplifier exhibits lower NEP since its voltage responsivity exceeds that of the detector without the preamplifier. 

**Figure 8:**
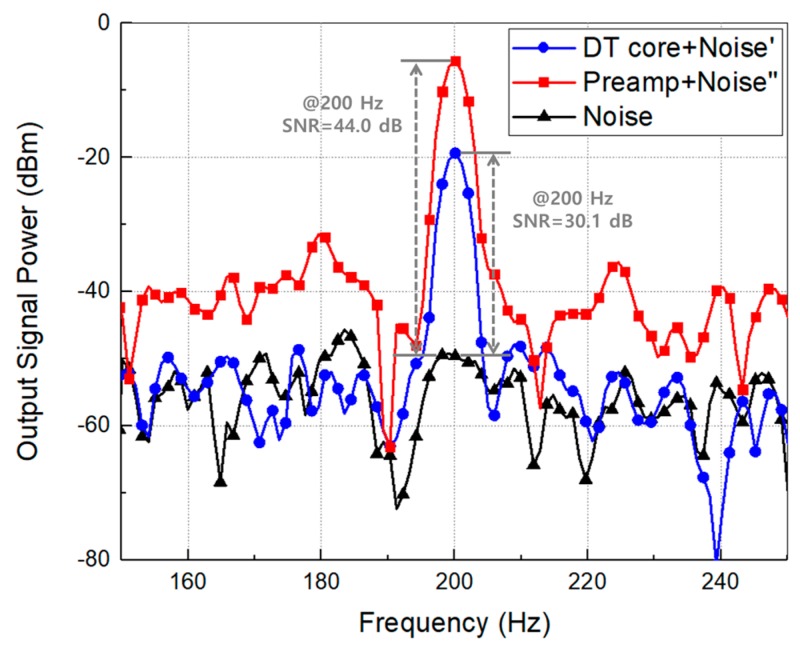
Signal-to-noise ratio at the outputs of the two detector integrated circuits using the modulation frequency of 200 Hz in the harmonic mixer.

Figure 8 shows the output power spectra of two THz detectors and the noise power. The modulation frequency of 200 Hz is used to decrease the effects on the noise of the power supply which are presented at 60 Hz and 120 Hz. The signal-to-noise ratios (SNR) of the two detectors at 200 Hz are measured to be 30.1 dB and 44.0 dB, respectively. The SNR improvement of 13.1 dB in the detector including the preamplifier is due to the increase of the voltage responsivity and NEP by using the preamplifier with the voltage gain of 1. The difference in the characteristics of the two detectors is caused by the presence or absence of the preamplifier at the output of the detector core. Since the preamplifier has a unity gain, it can be understood that the performance improvement of the detector including the preamplifier is not caused by the gain of the amplifier but the increase of the output impedance at the detector core by using the preamplifier. The measurement results between the two detectors, as summarized in Table 1, show that it is reasonable that the method to improve the detector performances based on the proposed analytical results in the fabricated CMOS plasmon detectors.

## 5. Conclusions

The performance analysis of the CMOS plasmon detector in the subthreshold region was examined by using the equivalent circuit model, which consists of superposition and small-signal methods. The superposition method explains the effect of the transmission lines at the input stage of the detector using the characteristic impedances. The plasmon detection at the subthreshold region was described by using the small-signal method. The analysis results expressed the voltage responsivity and the NEP of the detector as a function for the gate-to-source voltage. As a result of the analysis, the relationship between the input and output impedances of the detector and detector performance shows that the buffer amplifier at the output of the detector core increases the performance more than the characteristic enhancement by the gain of the amplifier. The detection characteristics and performance improvement were demonstrated in the measurement results of two fabricated detectors operating at 200 GHz. The proposed analysis shows that the method to improve the performances are presented for the CMOS plasmon detector by using the simple equivalent model.

## Figures and Tables

**Table 1:** Comparison of the CMOS detector performances

Ref.		Freq. [GHz]	CMOS Technology	Responsivity ^1^ [KV/W]	NEP [pW/$\sqrt{\mathrm{Hz}}$]
[20]		650	0.25 µm	80	300
[28]		856	65 nm	140	100
[29]		270	0.13 μm	300	18.7
[30]		365	90 nm	1200	200
This work	without preamplifier	200	0.25 μm	202	130
	including preamplifier	200	0.25 μm	482	39.3

^1^ Gate bias voltages and voltage gains of the main amplifier are different in each detector.
